# Preliminary study of the Craniofacial Pain and Disability Inventory-11: validation for patients with head and neck cancer

**DOI:** 10.4317/medoral.24673

**Published:** 2021-05-23

**Authors:** Beatriz Serrano-García, Isabel Bartrina-Rodríguez, José Manuel Zubeldia-Varela, José Luis Cebrián-Carretero, José Luis del-Castillo-Pardo-de-Vera, Joaquín Pardo-Montero, Alfonso Gil-Martínez

**Affiliations:** 1 Department of Physiotherapy, Centro Superior de Estudios Universitarios La Salle, Universidad Autónoma de Madrid, Spain; 2 Maxilofacial and Oral Surgery Department, Hospital Universitario La Paz, Madrid, Spain; 3 Motion in Brains Research Group, Centro Superior de Estudios Universitarios La Salle, Madrid, Spain; 4 CranioSPain Research Group, Centro Superior de Estudios Universitarios La Salle, Madrid, Spain; 5 Unit of physiotherapy, Hospital Universitario La Paz (IdiPAZ), Madrid, Spain

## Abstract

**Background:**

Cancer involves numerous physical, psychological and emotional changes and has a negative impact on patients. Although there are a wide variety of questionnaires for general use in patients with cancer, very few are available that assess the pain, disability and craniomandibular functionality of patients with head and neck cancer (HNC) in a more specific manner. The purpose of this study is to present the preliminary behavior of the CF-PDI in its reduced version adapted for patients with HNC.

**Material and Methods:**

A total of 61 patients with HNC were included in a study to preliminarily analyze the internal consistency of the instrument, the convergent validity and the floor and ceiling effects. All the patients completed the informed consent document and a battery of 5 questionnaires: The Numerical Rating Scale (NRS), the Tampa Scale for Kinesiophobia for Temporomandibular Disorders (TSK-TMD), the Pain Catastrophizing Scale (PCS), the Quality of Life Questionnaire in patients with HNC (QLQ-HN) and the reduced version of the Craniofacial Pain and Disability Inventory (CF-PDI-11). Patients also performed 2 physical tests: measurements of the pain threshold on the masseter muscle and on the distal phalanx of the first finger; and the maximum mouth opening in neutral head position.

**Results:**

Cronbach's α coefficient showed a very high internal consistency of 0.92. In terms of convergent validity, a statistically significant correlation was found between the CF-PDI-11 and the following variables: NRS, TSK-TMD, PCS, QLQ-HN, the threshold of pain in the distal phalanx of the first finger and the maximum interincisal opening. However, 21.3% of patients obtained the lowest possible score. The strongest correlation was found between the CF-PDI-11 and the QLQ-HN (r = 0.85, *p* <0.01).

**Conclusions:**

The preliminary version of the CF-PDI-11 shows that it could be a valid and reliable instrument to measure pain, disability and quality of life in patients with HNC.

** Key words:**Questionnaire, psychometric validation, head and neck cancer, pain, disability.

## Introduction

Head and neck cancer (HNC) includes multiple functional (e.g., swallowing, speech), physical (e.g., pain, structural changes) and psychosocial (e.g., social relationships, depression, anxiety) impairments (1). Many of these types of cancer arise in the head and neck region, with numerous anatomical locations and sublocations, among which are cancer of the lip and oral cavity, nasal cavity, paranasal sinuses, nasopharynx, oropharynx, larynx, hypopharynx, salivary glands and skull base (2). HNC is the ninth most common cancer in the world (3), and is significantly more common in men (2).

The main risk factors are tobacco use, alcohol consumption and viral infections, such as infection with human papillomavirus and Epstein-Barr virus, which correspond to oropharyngeal and nasopharyngeal cancer, respectively (2). Oropharyngeal cancer cases have been increasing, although with a higher survival rate, and are observed more frequently in those aged older than 50 years, with a greater number of new cases in men (4).

The loss of basic function generates disability in the patient and deterioration of their quality of life, limiting their food and drug intake as well as causing lifestyle changes, affecting their interpersonal relationships (5). Pain is typically the first symptom for consultation and is considered among cancer patients the alarm signal for which they go to the doctor. There is a broad symptomatology depending on the affected area, with underdiagnosed and undertreated pain (in different varieties and intensities) very present in all of them (6). Pain is typically a multifactorial experience, which complicates efforts to achieve adequate pain relief. Advanced HNC stages are often associated with pain, nerve damage and/or impaired function (7). Neuropathic pain is the most prevalent type in patients with cancer (8).

There is evidence of a significant association between psychological factors and cancer, with fear being a common problem among these patients. Furthermore, a strong association between poor quality of life and disability has been observed with higher levels of fear. Along these lines, an association was found between age, the presence and severity of physical symptoms (fatigue, pain and adverse effects of treatment) and certain psychological symptoms (anguish, anxiety and depression) (9). In addition, catastrophizing magnifies the severity and impact of pain as well as increases the fear of treatment failure, and a relationship between catastrophizing, depression and pain has been demonstrated (10).

Currently, there is a shortage of questionnaires in Spanish that assess pain, disability, and craniomandibular functionality in patients with HNC in a more specific manner, such as the Craniofacial Pain and Disability Inventory (CF-PDI) (11). However, its original design was focused on patients with chronic temporomandibular disorders and headaches. It is therefore necessary to develop new valid and reliable Spanish tools to specifically evaluate patients with HNC.

The purpose of this study is to present the preliminary behavior of the CF-PDI in its reduced version adapted for patients with HNC.

## Material and Methods

- Design

The study employed a preliminary validation design of the short version of the Craniofacial Pain and Disability Inventory (CF-PDI-11) for patients with HNC. This design aimed to verify the psychometric characteristics of the questionnaire in an initial sample of 61 patients from the Medical Oncology and Maxillofacial Surgery Department at La Paz University Hospital (LPUH).

The study followed the ethical procedures according to the rules of the Declaration of Helsinki and has the acceptance of the LPUH Ethics Committee for Research with Drugs (code: PI-1923). All patients signed the informed consent document before their inclusion.

- Sample description

The inclusion criteria were age older than 18 years and being diagnosed with primary HNC by a medical specialist. The exclusion criteria were presenting a traumatic history causing craniofacial pain, chronic craniofacial pain of another etiology, impossibility or difficulty in understanding the questionnaires, pregnancy, patients diagnosed with cancer other than HNC or a diagnosis of neurological diseases, chronic systemic diseases or previous rheumatic diseases.

- Procedure

After selecting the patients by means of a consecutive sampling, they were provided with all the necessary information and the informed consent was signed. A battery of questionnaires was completed and 2 physical examinations were performed. This battery of questionnaires included a sociodemographic information sheet and 5 evaluation questionnaires: the Numerical Rating Scale (NRS) (12), the Tampa Scale for Kinesiophobia for Temporomandibular Disorders (TSK-TMD) (13), the Pain Catastrophizing Scale (PCS) (14), the Quality of Life Questionnaire for patients with HNC (QLQ-HN) (15) and the CF-PDI-11 (Supplement 1). All the questionnaires, with simple closed-ended items, were self-administered. The physical tests consisted of pressure pain threshold measurements and maximum interincisal opening.

- Description of the instruments

Pain intensity: To measure pain intensity, the NRS was used, which consists of a 10-cm line, with values ​​from 0 to 10: 0 represents "no pain", and 10 "the worst pain imaginable" (12).

Kinesiophobia: The TSK-TMD assesses the fear of relapse of an injury in the craniomandibular area due to movement. Composed of 11 items, it consists of 2 factors: avoidance of activity and harm. It has been shown to have reliable psychometric characteristics (13).

Catastrophism: The PCS in its Spanish version seeks to measure the degree of catastrophizing associated with pain. It consists of 13 items and assesses 3 factors: rumination, magnification and hopelessness. Lower scores indicate less catastrophizing. It has been shown to have accepTable psychometric characteristics (14).

Quality of life: The QLQ-HN is one of the QLQ-Core-30 specific HNC modules used by the European Organization for Research and Treatment of Cancer. It consists of 35 items, with higher scores corresponding to a poorer quality of life. It presents 3 subscales, directed to the functionality, symptoms and global condition of the patient (15).

Development of the reduced version CF-PDI-11: The original CF-PDI consists of 21 items, with 4 multiple-choice responses, evaluating the factors of pain, craniofacial disability and functionality during activities of daily living (11). For this preliminary analysis of the CF-PDI-11, a reduced 11-item version of the original CF-PDI was employed.

To achieve the definitive reduced version, a panel of experts with experience in the development and validation of questionnaires in the field of pain and experts in cancer pain was created. The reduced version is made up of 11 items, having discarded some items from the original version (Supplement 1). The criteria for discarding the items were those questions that could not be answered by patients with HNC or that did not make clinical sense for these patients.

Pressure pain thresholds: Pain thresholds were measured in 2 locations: on the masseter muscle of the affected side (1.5 cm distal to the zygomatic arch and 2.5 cm anterior to the tragus) and on the first finger (dorsal part of the distal phalanx). A digital algometer (Wagner FDX 25) with an area of ​​1 cm2 was used, applying it with a pressure increase of 0.5 Kg/cm2/seg and perpendicularly (16). Three measurements were made with an interval of 30 seconds as indicated in previously described protocols (17).

Maximum interincisal opening: The maximum oral opening was measured in the neutral position of the head while recording the maximum opening measured from the lower edge of the upper incisors to the upper edge of the lower incisors (18).

- Statistical analysis

The statistical analysis was performed using the SPSS statistical program, version 20.0. The Kolmogorov–Smirnov test (over 50 participants) was used to verify the normal distribution of the variables. Descriptive statistics were presented as mean and standard deviation and maximum-minimum range for quantitative variables. The qualitative variables were presented by frequency and percentage. The statistical inference used to check the psychometric properties was performed using the following tests: (a) for reliability, internal consistency of the instrument was analyzed with Cronbach's α and the total correlation of items. Adequate internal consistency was considered when Cronbach's α was ≥0.70; (b) convergent validity between the CF-PDI-11 questionnaire and the TSK-TMD, PCS, QLQ-HN and its 3 subscales was assessed with Pearson’s correlation coefficient. The following correlations were considered: very weak between 0 and 0.3; moderate between 0.31 and 0.59; and strong from 0.60; (c) the ceiling and floor effects were analyzed according to the percentage of patients with the lowest (floor) and highest (ceiling) score in each dimension, considering that they are present if more than 15% of those surveyed achieved this score. Values ​​of *p*<0.05 were considered statistically significant. The confidence interval was estimated at 95%.

## Results

- Description of the study sample

The sample consisted of 61 patients diagnosed with HNC with a mean age ± standard deviation of 60.5 ± 12.5 years. The percentage of men was 67.2% of the sample. Regarding the level of study achieved by our patients, 6.6% had no previous studies, 21.3% primary studies, 34.4% secondary studies, 36.1% university studies and 1.6% data lost.

Regarding the treatment received for cancer, 49.2% did not receive any treatment, 19.7% received radiotherapy and 26.2% received radiotherapy plus chemotherapy. Related to employment status, 15% were active, 16.7% unemployed, 46.7% retired and 21.7% on leave due to disability. The descriptive statistics are shown in Table 1.

- Internal consistency

Cronbach's α coefficient presented a value of 0.92, showing a very high internal consistency. All the data related to the internal consistency of the questionnaires are presented in Table 2.

**Table 1:**
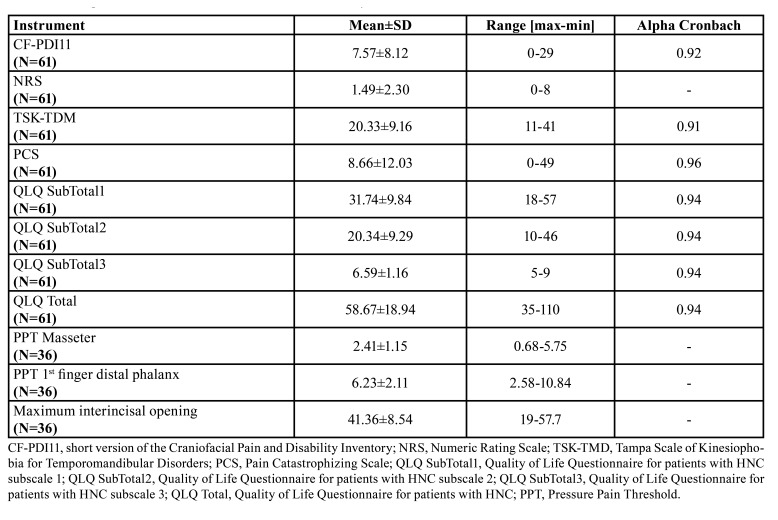
Descriptive statistics and estimates of internal consistency.

**Table 2:**
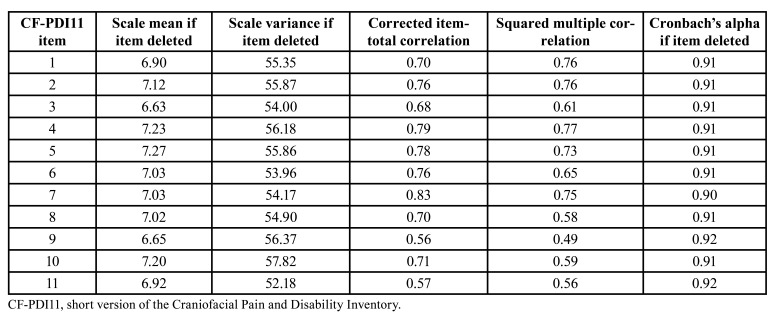
Corrected item-total between CF-PDI11 items.

- Convergent validity

Convergent validity was assessed with Pearson’s correlation coefficient. For this, the CF-PDI-11 was correlated with the TSK-TMD, PCS, QLQ-HN and its 3 subscales. All the data obtained statistically significant correlations with the CF-PDI-11. The strongest correlation was found between the CF-PDI-11 and the QLQ-HN (r = 0.85; *p* <0.01). The CF-PDI-11 was also significantly correlated with the pressure pain threshold, the maximum interincisal opening and the NRS, but not between the CF-PDI-11 and the pressure pain threshold in the masseter (r = −0.26; *p* >0.05). All the correlation data are presented in Table 3 and Table 4.

**Table 3:**
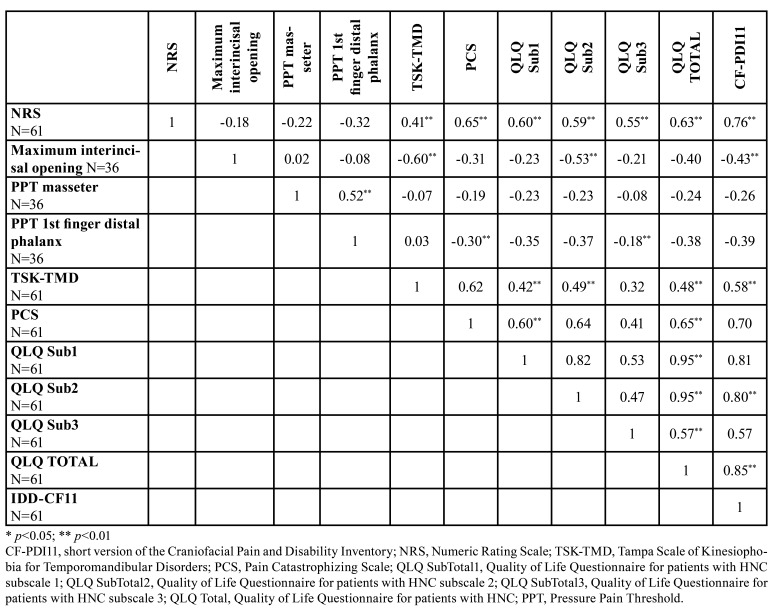
Pearson’s Correlation Coefficient of our main scales (convergent validation).

**Table 4:**
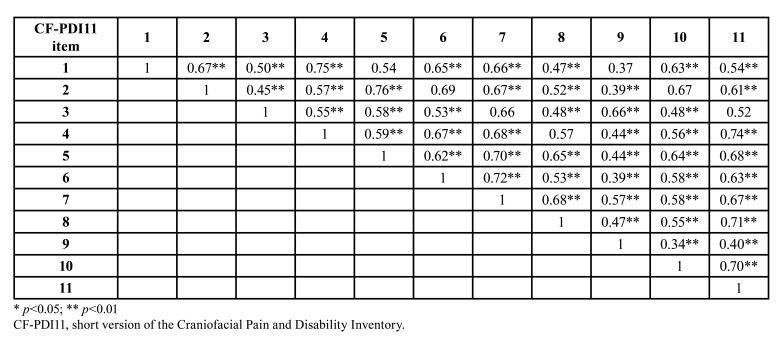
Correlation matrix inter-elements CF-PDI11.

- Floor and ceiling effect

No ceiling effect was identified in our sample; however, 13 (21.3%) of the 61 patients obtained the lowest possible score (0 points). The minimum and maximum ranges reported by the patients were 0 and 29 points, respectively.

## Discussion

The present study was designed to develop the reduced draft version of the CF-PDI-11 for patients with HNC. Specifically, internal consistency, floor and ceiling effect, as well as convergent validity were evaluated, demonstrating a fast, reliable instrument, without ceiling effect and with good convergent validity. It is an inventory that can be validated internationally, given it does not contain items specifically related to Spanish culture.

The study design had a biopsychosocial approach, because patients with HNC can present social and psychological factors caused by the anxiety related to their symptoms, diagnosis and the adverse effects of the treatment. The development of various inventories and questionnaires, such as the Cancer Needs Distress Inventory (19), in which the anguish of cancer patients is evaluated, or the Anxiety and Depression Scale (20), show that risk factors for depression throughout the evolution of the disease are related to the personality of the patient.

Various questionnaires are available to evaluate oncology patients in general; however, very few are specific for patients with HNC. Furthermore, a large number of the existing questionnaires are not validated in Spanish. Reviewing the available literature, we found certain questionnaires related to quality of life, such as the Functional Assessment of Cancer Therapy, Head and Neck (21) and the Oral Health Impact Profile-14 (22) as well as the Liverpool Oral Rehabilitation Questionnaire (23), which focuses on the impact of rehabilitation in these patients. On the other hand, the Brief Core Set Questionnaire (24), which is the reduced version of the University of Washington Quality of Life Questionnaire (25), is validated in Spanish; however, it is an excessively long questionnaire compared with the CF-PDI-11. Regarding disability, we found the Carcinologic Handicap Index (26), and in relation to patient concerns, we found the Patient Concerns Inventory-Head and Neck (27). Therefore, the CF-PDI-11 could be the first reduced instrument in Spanish to assess some factors related to patients suffering from HNC.

Our sample consisted of a higher percentage of men (67.2%), which coincides with the literature that shows a higher prevalence in males. This higher prevalence could be associated with a greater amount of substance abuse by men, such as alcohol and tobacco, although the HNC caused by this is decreasing due to increasing awareness of the harmful health effects of these substances (28). On the other hand, the overall rate of HNC is increasing due to the human papilloma virus due to the sexual behaviors of the population, with an increase in oral sex and the number of sexual partners. This would not fully explain the higher prevalence in men.

On the other hand, 49.2% of the patients were not receiving treatment, given their recruitment was performed prior to treatment initiation. We should therefore consider that the literature indicates that both pain and disability often manifest themselves after the completion of treatment in these patients (29).

- Associations

The CF-PDI-11 showed important associations with other questionnaires and tests performed, except in the pressure pain threshold on the masseter muscle. The majority of these associations was observed between the CF-PDI-11 and the QLQ-HN in quality of life. It should be considered that beyond the mortality that patients with HNC present, quality of life is by far the most valued variable (15), which is clinically key in these patients’ perception of well-being.

A strong association with fear has also been observed. This fear is especially present in the young population, in those who are undergoing complex surgery, chemotherapy or radiotherapy treatments and in those who in most cases believe that there will be a recurrence at some point in their life; so much so, that there are even specific questionnaires to assess fear of recurrence (30).

- Clinical implications

In patients with HNC, physical and psychosocial conditions of various kinds develop, with pain, disability and dysfunction throughout the evolution of the disease. Unfortunately, there is evidence of insufficient pain management in these patients; thus, the development and use of inventories such as the CF-PDI-11 are necessary to achieve a better approach by measuring and recording the variables involved in this disease.

The authors of the present work consider that the CF-PDI-11 could become a valid and reliable instrument in the Spanish-speaking population to measure the variables of pain, disability, functionality and/or quality of life in patients with HNC. In addition, the CF-PDI-11 aims to cover aspects that the patient demands in a fast and efficient way, given the time of its completion is estimated at approximately 1 minute and 30 seconds.

- Limitations

First, this is a preliminary study; although the development of a study with these characteristics could be promising and of interest, it has some limitations associated with the size of the sample. That is why we were unable to perform a factor analysis of the instrument. For a definitive sample size, at least 100 participants will be necessary for a factor analysis to be performed with sufficient guarantees in accordance with data stability and variability criteria. Furthermore, various authors have suggested that a sample of 10–15 participants per item might be adequate for this type of statistical analysis. In this case, 100 to 110 participants would be necessary.

On the other hand, there was no reevaluation to check the reliability of repetition by means of the Intraclass Correlation Coefficient by means of test-retest, given a large portion of the patients were about to begin treatment and their responses might be different after the intervention. We must also emphasize that a considerable number of patients did not want to participate in the study due to their poor general condition due to the disease itself. Also, it should be considered that the sample was made up of patients with a variety of HNCs and therefore they had differing characteristics. Cronbach's α coefficient was quite high in its result, which, although it is still too early to determine, could suggest the possibility of some redundant questions. Furthermore, although the study is preliminary, another limitation could be the appearance of a 21.3% floor effect. In the definitive study, it will be necessary to determine whether any of the questions did not apply to a significant part of the population and whether the selection of the sample was optimal.

Future research with a minimum sample of 100 patients would allow a factor analysis and validation of the usefulness CF-PDI-11 in patients with HNC.

## Conclusions

The preliminary version of CF-PDI-11 shows that it could become a promising, valid and reliable instrument for use in patients with HNC. This preliminary questionnaire has shown a high association with the QLQ-HN of quality of life in patients with HNC. A future study with more than 100 participants is required for its final validation.
